# Can digital brain twins dissolve the uncertainties surrounding unresponsive wakefulness?

**DOI:** 10.1186/s12910-026-01390-x

**Published:** 2026-01-30

**Authors:** Giuseppe Comerci, Stella Mosetti, Andrew J. Barnhart, Matthias Braun

**Affiliations:** 1 Department of Social Ethics, University of Bonn, Bonn, 53113 Germany; 2 Interdisciplinary Centre for Health Science, Sant’Anna School of Advanced Studies, Pisa, 56127 Italy

**Keywords:** Digital brain twins, Unresponsive wakefulness syndrome, Uncertainty, End-of-life, Ethics

## Abstract

Unresponsive Wakefulness Syndrome (UWS), a condition characterized by wakefulness without awareness, presents significant medical and moral uncertainties, particularly in end-of-life decision-making. Digital Brain Twins (DBTs), virtual replicas of patients’ brains driven by advanced artificial intelligence, offer the potential to alleviate medical uncertainties by providing precise diagnoses, prognoses, and experimental platforms for treatment testing. This paper provides a theoretical contribution by examining the potential ethical impact of these technologies in the context of UWS. We argue that, while DBTs promise greater diagnostic accuracy, personalized predictions of recovery, and non-invasive tools for exploring therapeutic interventions, they do not necessarily resolve the moral uncertainties faced by proxy decision-makers. Decisions about withdrawing or continuing life-sustaining treatment are fundamentally moral and value-laden, often extending beyond empirical evidence provided by DBTs. Factors such as cognitive biases, emotional distress, and subjective interpretations of best interest further complicate these decisions. While DBTs represent a breakthrough in precision medicine, their role in navigating the ethical complexities of UWS is limited, emphasizing the need for integrated approaches that combine technological innovation with ethical and psychological support for proxies.

## Introduction

Among the various neurological conditions that challenge modern medicine, *Unresponsive Wakefulness Syndrome* (UWS), also known as *vegetative state*, stands out as a condition fraught with ambiguity.^1^Unresponsive Wakefulness Syndrome (UWS) is a medical condition within the spectrum of disorders of consciousness, typically categorized alongside coma and a minimally conscious state [3, 4]. UWS results from severe brain damage due to trauma or anoxia [3, 5]. Patients with UWS exhibit wakefulness without awareness characterized by open eyes and sleep–wake cycles but do not respond appropriately to stimuli, resulting in a lack of behavioral evidence of consciousness [3]. UWS can last for a few weeks (*persistent UWS*) or, in some cases, indefinitely (*permanent UWS*) [6].

UWS inherent characteristics, its boundaries and progression are often unclear, and our understanding of this condition remains limited [7, 8]. These features create significant uncertainty in clinical management. As a result, UWS is characterized by a certain degree of medical uncertainty, which encompasses a lack of reliable information about diagnosis, prognosis, and treatment options from both the physician and patient perspectives [9]. This uncertainty also extends to the moral dimension of the condition. Indeed, UWS frequently raises complex ethical issues, particularly in end-of-life decision-making [10–12] Given that patients with UWS cannot express their preferences, a proxy—typically a close family member—is appointed to make decisions on their behalf [13]. However, without advance directives, proxies might not have essential information, further complicating the decision-making process. More accurate clinical assessments can provide valuable insights into the patient’s current state, prognosis, and possible treatments.

At the same time, emerging technologies based on artificial intelligence (AI) are increasingly presented as tools that could alleviate these uncertainties [14, 15], one of which is the development of *digital brain twins* (DBTs). This technology generates a virtual dynamic model of a patient’s brain by leveraging structural and functional data from the patient, which can be used for personalized diagnosis, prognosis, and pharmacological testing [16, 17]. To the best of our knowledge, DBTs to deal with UWS cases are not yet developed; indeed, in principle DBTs could be used to distinguish UWS from other forms of disorders of consciousness, refine the trajectories of the condition and possible recover, and to safely test possible pharmacological interventions for the patient *in silico.* DBTs, then, are entering precisely a clinical space where uncertainties around the medical and moral aspects of care are acutely charged.

This dual development of both the persistence of deep uncertainty in UWS and the rise of powerful AI-driven tools such as DBTs matters for at least two reasons. First, the ethical stakes in UWS are exceptionally high. Decisions about continuance or withdrawal of artificial nutrition and hydration, intensive care, and experimental interventions determine whether a patient lives for years in a UWS state, dies sooner, or is exposed to novel therapies with uncertain benefits or harms. Small shifts in diagnostic or prognostic judgements can dramatically change how proxies and clinicians interpret a situation and what they find as ethically permissible or obligatory.

Second, DBTs thus fit within a broader precision and predictive medicine narrative that implicitly or explicitly promises not only better data, but better decisions. In disorders of consciousness, this can easily slide into the thought that once the medical uncertainties are greatly reduced, the moral uncertainties surrounding patient care will become clearer. Clarifying what DBTs can and cannot do in relation to both medical and moral uncertainty is therefore crucial. If their outputs are taken to dissolve moral disagreement, or to settle contested questions about best interests and end-of-life choices, then their epistemic limitations and the value judgements built into their use may be obscured. Conversely, if their role is more modest, then we need a framework for understanding how better empirical knowledge interacts with, but does not replace, moral reasoning.

This paper examines the relationship between medical and moral uncertainty in UWS, along with how DBTs fit within this context. Thus, the following question arises: does reducing medical uncertainty through DBTs also lead to a greater reduction of moral uncertainty for proxies in end-of-life cases involving UWS? We argue that while DBTs may significantly reduce medical uncertainty, they do not necessarily translate into a reduction of moral uncertainty for proxy decision-makers. To do so, we philosophically analyze the theoretical impact of DBTs on medical and moral uncertainty, respectively. We first conceptually ground medical and moral uncertainty and how this will further frame our argument. Second, we examine how these uncertainties present themselves in UWS contexts. Third, we introduce DBT technology and explore its potential for reducing medical uncertainty. Finally, we discuss why improved medical information does not automatically equate to greater reduced moral uncertainty for proxy decision-makers.

## Uncertainty terminology and concepts

Uncertainty is a complex concept with no universally shared definition, and its meaning varies depending on context. From an epistemological perspective, uncertainty occupies the middle ground between fully informed knowledge and total ignorance. Thus, uncertainty entails a partial lack of information or information insufficiently precise to provide a clear understanding of a given circumstance. Here, we examine how uncertainty manifests specifically in medical and moral contexts, setting the stage for our analysis.

Uncertainty is widely recognized as an inherent part of medicine [18] and is often reported as a “ubiquitous” element in clinical practice [19, 20]. Indeed, the complexity of the human body, with its numerous microscopic and macroscopic interactions, makes medical certainty difficult. Even expert physicians often encounter cases without clear answers. However, uncertainty in medicine has been analyzed extensively, ranging from the patient’s perspective [21], which focuses on specific clinical problems [22], to broader frameworks [23]. For our purposes, the framework provided by Han and colleagues is particularly useful, as it systematically categorizes uncertainty in healthcare into distinct domains [9]. Based on extensive literature review, Han et al. identify three domains of uncertainty: (1) scientific uncertainty (disease-centered), (2) practical uncertainty (system-centered), and (3) personal uncertainty (patient-centered). Each domain is further divided into more specific subcategories. However, our analysis focuses primarily on scientific uncertainty, as we are concerned with uncertainty arising from medical data related to specific conditions. Scientific uncertainty encompasses four subdomains: diagnosis, prognosis, causal explanation, and treatment recommendations. This categorization clarifies precisely what aspects should be considered in the management of uncertainty during clinical practice.

Having outlined uncertainty in the medical context, we now turn our attention to moral uncertainty. Hicks describes moral uncertainty as “… a[n] [epistemic] state in which one’s credences are split between mutually exclusive *moral* propositions” [24]. According to Hicks, the source of these conflicting propositions—and, consequently, the uncertainty itself—could be either a moral (normative) or a non-moral (descriptive) claim. To illustrate Hicks’ distinction, consider the following scenario: suppose a woman is walking in the countryside and suddenly feels hungry. She notices a mansion nearby with an apple tree laden with fruit. If her uncertainty revolves around whether she can sneak into the garden unnoticed, she would face a descriptively-based moral uncertainty––which depends on factual circumstances. However, if she questions whether it is morally acceptable to trespass and take an apple, her uncertainty is a morally-based moral uncertainty––it involves conflicting moral propositions. This distinction between descriptive and normative sources of uncertainty will be important for our subsequent analysis.

## Unresponsive wakefulness syndrome and uncertainty

### Scientific Uncertainty in UWS

Diagnosing UWS can be a difficult phase of its management. Studies report substantial diagnostic errors owing to overlapping symptoms with other conditions of consciousness such as minimally conscious states or locked-in syndrome [25]. One study reported that 43% of 40 patients with UWS were misdiagnosed [26]. A patient in a minimally conscious state can show minimal awareness of their surroundings, spontaneously move their eyes, swallow autonomously, or respond properly to external stimuli [27]. Accidental movements in UWS patients can appear intentional, leading clinicians to misinterpret the patient’s level of awareness. Furthermore, recent work on covert awareness has shown that some patients who meet behavioral criteria for UWS nonetheless display evidence of preserved conscious processing when assessed with advanced neuroimaging and electrophysiological paradigms [28–30]. Such diagnostic uncertainty can profoundly impact subsequent treatment decisions.

The prognosis of UWS patients similarly involves considerable uncertainty. Currently, some criteria help predict the possible evolution of UWS over time. The chances of recovery largely depend on the severity of the trauma, and many patients who do recover emerge from UWS within a few weeks, with better outcomes for younger patients [31]. Patients who remain in UWS for six months after the initial trauma have lower chances of recovery [32], and these chances decrease even further after one year [33]. However, these criteria provide only general indications. Assessing the possible outcomes of UWS requires estimating the extent of neurological damage if the patient regains awareness and then hypothesizing their future quality-of-life. Patients may, for instance, transition to a minimally conscious state rather than full awareness, making prognosis even more uncertain.

In contrast, causal explanations for UWS typically involve well known mechanisms, such as traumatic or anoxic brain injury, thus involving minimal uncertainty [5]. These mechanisms are broadly understood, and extensive clinical evidence supports their role in causing UWS. Consequently, compared with diagnostic or prognostic assessments, determining the primary cause of a patient’s UWS rarely poses significant challenges for clinicians.

Treatment recommendations, however, do hold uncertainty as well. There is currently no known method to induce a patient to emerge from the UWS and regain consciousness, and efforts remain focused on supportive care. However, several approaches have been tested, ranging from pharmacological therapies [34], experimental approaches with psychedelics [35], to surgical interventions [36]. Nevertheless, running clinical trials with UWS patients is ethically challenging. As with other experimental interventions in medicine, such studies should be conducted under conditions of clinical equipoise [37]. However, in UWS the combination of small, highly vulnerable patient population, reliance on surrogate consent, and a strong imperative to minimize risk typically results in few, small, and methodologically limited trials. Consequently, substantial residual uncertainty about the benefits and harms of potential treatments persists, and the number and intensity of interventions that can be justifiably tested in these patients is sharply constrained.

### Moral uncertainty in UWS

Having explored medical uncertainties, we now consider moral uncertainties arising in UWS, especially in end-of-life decisions. UWS is frequently associated with moral dilemmas regarding whether to continue or withdraw life-sustaining treatments such as artificial nutrition and hydration. In jurisdictions where withdrawal is permitted, such an option becomes viable when UWS persists without signs of improvement. Since patients with UWSs cannot participate in decision-making, proxies (usually close family members) must decide on their behalf. Ideally, explicit advance directives would simplify this decision-making process by clarifying patients’ preferences and thereby increasing moral certainty about end-of-life choices. In practice, however, advance directives are often missing, outdated, or vague [38–40], which introduces substantial moral uncertainty into the decision-making process. As outlined above, Hicks distinguishes between descriptively-based and morally-based uncertainty [24]. In UWS cases, descriptively-based moral uncertainty arises when proxies are unsure about factual matters that are crucial for moral evaluation. Such matters can include the likelihood that the patient will recover consciousness, the extent of residual neurological damage if recovery occurs, or the patient’s expected quality of life. When a proxy lacks clear and reliable information about these facts, they may be unsure which course of action would best respect the patient’s interests or previously expressed values.

By contrast, morally-based moral uncertainty concerns ambiguity or conflict about the relevant ethical norms, even when the empirical facts are relatively clear. Proxies may be uncertain, for instance, whether it is morally permissible to withdraw artificial nutrition and hydration from a loved one who is unlikely to recover, or whether they are instead morally required to preserve biological life for as long as possible. Such uncertainty may arise from tensions between religious commitments, legal norms, cultural expectations, and personal moral intuitions.

Although morally-based moral uncertainty appears to have normative primacy while deliberating, descriptively-based moral uncertainty often exerts a dominant influence in practice. For many proxies, the pressing question is: “Given the data I have about the patient, is it morally acceptable to withdraw artificial nutrition?” When scientific uncertainty about diagnosis, prognosis, or treatment options is high, it is difficult for proxies to answer this question confidently, even if their underlying moral framework is relatively stable. As Zilio emphasizes, the opacity of the empirical situation in disorders of consciousness can itself generate or amplify ethical uncertainty [41].

This interplay between scientific and moral uncertainty is central to our subsequent discussion. A technology such as a DBT promises to reduce scientific uncertainty by improving diagnosis, prognosis, and treatment simulation. At least on the surface, this might seem to offer a pathway to greater moral certainty for proxies. In the remainder of this work, we argue that although DBTs could substantially reduce descriptively-based moral uncertainly, they cannot, by themselves, resolve morally-based moral uncertainly. Decisions about withdrawing or continuing life-sustaining treatment in UWS remain fundamentally moral decisions that extend beyond what even the most precise empirical predications can determine.

## DBTs: a valuable medical tool

A digital twin (DT) is an AI-driven technology that digitally simulates an object or process in real-time, aiming to monitor and predict its future state. Initially developed in the industrial sector to optimize product life cycles [42], this technology has recently attracted significant interest in precision medicine. For example, recent work on cardiac digital twins has shown that personalized, physics- and physiology-constrained in silico hearts can be generated from clinical imaging and electrocardiographic data to uncover patient-specific electrophysiological mechanisms and support individualized decision-making in ischemic heart disease and arrhythmias [43, 44]. By creating a virtual replica of a patient’s organ, DTs can enhance diagnosis, suggest therapies, predict prognoses, serve as platforms for drug discovery, and enable increasingly personalized care. Similarly, a DBT could significantly reduce medical uncertainty in managing UWS. DBTs could provide accurate predictions of disease progression and potential quality-of-life outcomes if recovery occurs.

One of the most interesting features of DT technology is its dynamic representational properties. Unlike static anatomical images, a DBT continuously mirrors the patient’s brain in real-time. Any physiological change in the patient’s brain is reflected in the DBT, maintaining a precise one-to-one correspondence. Moreover, DBTs can simulate not only the current state of the brain but also predict its future states. This is possible because machine-learning and advanced artificial intelligence algorithms cross-reference data from an individual patient with the data sourced from the broader patient populations who share the same medical condition. In this way, DBTs offer highly personalized and statistically robust predictions, making them potentially highly valuable tools for clinical decision-making in UWS patients.

Simulating the brain, however, remains a significant scientific and computational challenge. Over recent years, several ambitious projects have attempted to simulate specific brain regions [45] or even large-scale simulations of the entire brain. In this latter case, a significant recent attempt has been made by the *Human Brain Project* [46], which paved the way for other relevant projects that developed DBTs for medical implementation. Among these projects deserved to be mentioned the *Neurotwin* project [47], which aimed to employ DBTs to improve neudomodulation therapies in patients affected by Alzheimer’s disease; the *Gemini project* [48], that through personalized brain simulations deals with brain stroke; and the recent *Virtual Brain Twin* project [49], which aims to extend the scope of use of DBTs to enhance the treatment of mental disorders. Despite the increasing interest in DBTs, to the best of our knowledge, no concrete proposals have been made yet to develop DBTs to address UWS cases; however, some papers have already highlighted the advantages of using large-scale brain simulations for disorders of consciousness, emphasizing the potential of these simulations to better explore and understand the concept of consciousness, as well as anticipate several useful applications of this technology in clinical practice [50, 51]. Given the urgency of UWS-related ethical and medical challenges and the rapid advancement of computational neuroscience, developing DBTs tailored to UWS may soon become feasible. In the following paragraphs, we conceptually illustrate how DBTs may contribute to reducing the scientific uncertainty related to UWS.

Accurate diagnosis is crucial in UWS management, as misdiagnosis can significantly impact treatment decisions and prognostic assessments [26]. DBTs could potentially enhance diagnostic accuracy by integrating patient-specific brain data with extensive datasets from other patients [16]. This cross-referencing capability would allow clinicians to better distinguish UWS from similar conditions, such as minimally conscious states or locked-in syndrome. Additionally, DBTs could serve as retrospective diagnostic tools, verifying or challenging previous clinical assessments. Furthermore, in clinical practice, seeing a body is pivotal for assessment and diagnosis. This epistemic clarity is often achieved at the cost of invasive procedures that expose the organ and the patient to further risk. Unlike invasive diagnostic procedures, DBTs offer a non-invasive, detailed, and dynamic view of the patient’s brain, providing clinicians with valuable insights into additional risks.

Regarding prognostic uncertainty, current prognostic methods, including functional neuroimaging [52], evoked potentials [53], and machine learning [54] have shown promise but remain limited. DBTs could substantially improve prognostic accuracy by providing personalized predictions of recovery likelihood and potential quality-of-life outcomes. Indeed, in UWS some unexpected recovery can occur, as reported in the literature [55]. By integrating individual patient data with broader population datasets, DBTs could theoretically identify rare recovery scenarios typically overlooked in clinical practice.

Finally, DBTs may improve to reduce the uncertainty around treatment recommendations, providing an experimental platform for testing novel therapeutic interventions. A DBT offers the opportunity to bypass certain ethical issues related to experiments with UWS patients. Indeed, it is possible to dispose of a tool where there is no limit to the number of tests that can be run. Every medical intervention can be simulated in a safe environment without any consequences for the patient, and the results obtained will have a high degree of truthfulness owing to the precise simulation of the patient’s brain characteristics. This aspect of DBTs allows for reducing possible side effects, refining the interventions before administration, and ensuring a personalized solution.

Notably, the DBT considered in this work is limited to a virtual replica of the features and functions of the physical brain. Thus, the simulation does not imply any psychological traits of the patient; therefore, no personal preferences or directives can be extrapolated from the replica. Over the years, some proposals to infer the preferences of incapacitated patients have advanced. This is the case with the *patient preference predictor* proposed by Rid and Wendler [56], which, based on surveys of a large population about their preferences in hypothetical clinical situations, along with the collection of numerous demographic data, would lead to the development of an algorithm capable of returning the putative preferences of a specific patient. This approach is further explored by Earp and colleagues, who proposed a *personalized patient preferences predictor* (P4) [57]. P4 technology takes the form of a fine-tuned large language model trained on text produced by the patient himself and capable of providing his personal preferences in end-of-life situations [58]. Even if P4 technology has been criticized for both its technical feasibility [59] and reliability [60], the reason why it is not considered in this paper is that the aim of this work is not to examine putative preferences related to an incapacitated patient but rather to assess the possible impact of a tool capable of predicting his or her physical condition on moral (un)certainty.

## More data, more moral certainty?

DT technology for medical purposes has sparked a lively discussion about its ethical implications [61–63]. In particular, Lupton highlighted the importance of the language used to describe this technology [64]. She argues that the metaphor implied by the term *digital twin* conveys a false promise; the term is misleading, as the word *twin* suggests a strong resemblance. In reality, DTs used in medicine are only approximated models of the real entities they aim to replicate, exhibiting limited fidelity. This may generate unrealistic expectations among the general public or influence the allocation of research resources. To address this ethical concern, Lupton suggests adopting alternative terminology such as *simulation* or *computerised model* [64]. This problem affects DBTs even more acutely, as the brain is among the most complex entities in the known universe. Evers and Salles [65] illustrate this issue by identifying two major challenges in building a DBT: ontological complexity, referring to the physical complexity of the object to be twinned, and epistemic transparency, referring to the extent and clarity of our knowledge about that object. In this light, while the heart is relatively well understood in terms of its structure and function, making the construction of a reliable cardiac digital twin feasible, the brain presents an extraordinarily high level of ontological complexity, and our knowledge of it remains limited. Consequently, DBTs are far from being faithful replicas of a patient’s brain, and the term “twin” can therefore be considered misleading.

The issue is not only linguistic but also encompasses technical and practical aspects. Indeed, given the approximations inherent in the replicated brains, the reliability of DBT outputs could be questioned, particularly when applied to complex decision-making cases in UWS. To address these limitations, current DBTs represent a compromise between spatial resolution and biological realism [65]. They aim to maximize the fidelity of simulations in those brain regions that are most relevant for a specific case. Therefore, a DBT does not need to be a full-scale simulation of an entire brain; rather, given the issues mentioned above, it should be as simple as possible and as complex as necessary [66]. In this way, unreliability can be minimized, thereby making DBTs worth using.

However, when information obtained through DBT is used to make moral decisions about patients in a UWS, the application of this technology reveals several shortcomings. To illustrate these limitations, we develop a two-fold argument in the following sections.

### Between what we know and what we ought to do

The core question of this paper is whether having more and better information obtained through a DBT about the clinical condition of a UWS patient could be enough to dissolve the moral uncertainty related to that condition. This issue is prompted by medical approaches such as *evidence-based medicine*, which relies on the use of the best current evidence to inform medical decisions. The clarity of such evidence can be achieved by employing advanced methodologies and techniques that yield valuable information. Similarly, moral decision-making should be grounded in the best and most relevant medical evidence available. Evidence-based medicine thus regards the reduction of ignorance as a moral imperative, as it leads to better-informed and more justified decisions [67]. This, in turn, requires consideration of all relevant and available medical facts to avoid compromising the plausibility of moral reasoning [68].

Against this background, our question about moral certainty through data precision became relevant. To better grasp the issue in question, consider the following fictitious scenario:

*H is the husband of a woman (W) with UWS. W has always been a fervent believer and practitioner of a religion that considers the withdrawal of artificial nutrition to be a form of killing and*,* therefore*,* an act that contradicts her credo. By putting himself in the shoes of W*,* H is willing to endorse this religious dogma and keep her connected to life support. However*,* a DBT developed from W’s brain predicts no chance of recovery but also indicates that the patient has been*,* and will continue to be*,* in constant pain. What should H do?*

This case aids in discussing the reasons behind our thesis. How could the role of DBT be assessed in this case? Would having a digital copy of W’s brain help H make the best decision for her? We argue that the answer to this question is negative, and the reasons are essentially twofold.

First, while DBTs could enhance empirical understanding, they do not determine moral decisions. While highly precise and reliable empirical data about the patient’s condition are available, this information alone does not indicate which course of action is morally preferable. The fact that patient W is suffering and will not recover does not automatically imply that treatment should be withdrawn. There is no logical connection between describing the clinical state of W and determining what H ought to do. This problem is well known as *is–ought problem* or the *Hume’s fallacy*. Hume’s fallacy highlights the inherent risks and methodological tensions involved in any attempt to extract substantive normative conclusions from empirical evidence. This risk becomes particularly evident when we consider DBTs. Considering the epistemic and ontological limitations of the very concept of “twin” highlighted above, a DBT does not replicate a patient as a whole but offers a model-based simulation of his/her brain’s physiological state. This epistemic gap (between the individual and the computational representation of the brain) raises moral questions. A DBT conveys the impression of providing an unprecedented level of empirical detail regarding neural states, prognostic indicators, pain correlates, likelihood of recovery, and related parameters. However, knowing everything about a person’s brain will not tell us who that person was. A DBT tells us very little about that person’s interests, about what they would have wanted for themselves, and, in the case of W, about the extent to which her moral convictions outweigh her suffering.

### Knowing interests vs. deciding which interests count

This brings us to the second reason why having more information about a person’s brain will not resolve moral uncertainty. Indeed, the use of DBT in these cases could potentially show a conflict between *descriptively-based* and *morally-based* moral uncertainty, potentially more than other medical technologies. Indeed, the conflict arising from the use of DBTs is not only about the relationship between empirical facts and moral principles, but also about which facts are morally relevant in determining the patient’s best interest. Wilkinson [69] describes an interest as “having a stake in something — that is, standing to gain or to lose, depending on the nature or conditions of that something”. This “something to gain or lose” necessarily refers to something from which we can either benefit or be harmed, highlighting that interests are always tied to potential effects on our well-being. Precisely for this reason, the principle of best interest is generally interpreted in a consequentialist and maximizing framework [70]. This principle is defined by Buchanan and Brock as “acting so as to promote maximally the good of the individual” [71]. However, when discussing patients’ well-being, or describing what is “good” for someone, challenges arise because defining it is not always straightforward, particularly in end-of-life care.

As noted, in UWS cases *descriptively-based* moral uncertainty arises when proxies are unsure about factual conditions that are crucial for making moral evaluations. Such factors include, for instance, the likelihood of recovery, the patient’s quality of life, and the possibility that the patient is experiencing suffering. A DBT could potentially provide information on all these aspects. However, this information alone may not be sufficient to determine the patient’s best interests, highlighting the presence of *morally-based* moral uncertainty.

Thinking about H, it can be inferred that before the DBT prediction, it would have been easy for him to identify the best interest of W with the adherence to her beliefs; however, the new information provided makes the best interest even more blurred. Indeed, what should be the best interest for W? Some critics have argued that there is no objective best interest and that death itself cannot fall within a best interest scenario [72]. While a DBT could guide us in choosing different treatment courses, it does not indicate which path should be prioritized in line with the UWS patient’s best interest. It is uncertain whether H should act with his W’s beliefs related to end of life in mind or consider a broader set of interests that may concern all patients in general such as the avoidance of pain. In fact, the principle of best interest seems to allow any arbitrary set of factors to be determined in the evaluation [73], and it is not even certain that the patients’ “objective” best interest aligns with what they would want for themselves, or if following their preferences means genuinely acting in their best interest. This expresses *morally-based moral uncertainty*. Indeed, while a DBT can give us more factors on which to base our decision, it cannot tell us which factors should prevail in the final moral decision- Paradoxically, the more data we have, and thus the more descriptively-based uncertainty is reduced, the more moral uncertainty seems to increase.

One way in which this conflict can manifest in clinical practice is also between physicians and the patient’s caregivers. There may be a deep moral disagreement between W’s husband and the medical team about keeping a person who is suffering alive. A physician may feel a moral duty to end an irreversible situation that only causes the patient’s pain; a husband who knows the patient, on the other hand, may feel obliged to fully respect her beliefs regarding her spirituality.

Another case that can support our thesis is the following:

*S is the mother of a 5-year-old son who*,* as a result of a traumatic brain injury from an accident*,* now has UWS. Given the child’s young age*,* S has no idea about her son’s possible preferences in such a situation. S has the option to request the withdrawal of artificial nutrition but feels unable to choose. A DBT of the child’s brain is then developed and implemented to bring clarity to the situation. The DBT predicts a 40% chance of recovery*,* but this probability is associated with a severe cognitive disability that will probably affect the child for the rest of his life.*

In this case, we have no evidence that the patient is experiencing pain. However, the issue of disability may still carry weight in the mother’s decision. There are well-known cases that have sparked reflections on whether death may be in the best interest of a child with a cognitive disability [68] or a child who is seriously ill [74]. We believe that a definitive universal answer to these debates is impossible to achieve. What is unlikely to end, instead, is precisely the moral disagreement surrounding these cases, which is sometimes brought before the courts [75].

Beyond the relationship between *descriptively-based* moral uncertainty and morally-based moral uncertainty, it is important to highlight that the proxy’s decision to withdraw from treatment is undoubtedly a moral decision, and our moral decisions rarely depend solely on empirical data. While there is no doubt that using such data the epistemic position of decision-makers improved, an ethical decision involves a set of values and beliefs that may have nothing to do with the value of a prediction or a set of more accurate data [76]. Many values, such as the dignity of patients, their quality of life, and avoiding pain or suffering, have been identified as factors that patients and proxies consider when making treatment decisions. Nevertheless, the consideration of these values depends little on the validity of the prediction and diagnosis that a DBT could offer. Moreover, we know that even when the patient’s wishes are clearly known and expressed through advance directives, proxy decision-makers may choose to act differently from what the patient has stated. This also occurs because when considering morally complex choices such as the suspension of treatments that cause the death of a patient, some cognitive and psychological mechanisms can come into play, which could unintentionally lead to the patient will not being respected. Research shows that *status quo* bias can have a meaningful effect on the proxy’s decision; people are in fact psychologically uncomfortable with change [77], and they tend to prefer maintaining the current situation over changing it. This is also associated with a preference for an error of omission rather than an error of commission [78]. This can lead to, for example, a moral surrogate deciding not to suspend treatment for their loved one in a UWS, even if the patient might have preferred otherwise, and even if the DBT tells us that the chances of recovery are null. Furthermore, research has shown that proxies’ decisions in end-of-life scenarios often depend on incorrect assumptions, which are also due to the emotional distress of the situation. The similarity fallacy, namely, the false belief that the patient thinks in the same way as the proxy, represents a potential risk, along with a whole series of other misjudgments that reflect the psychological difficulty of the moment. This happens because we assume that those closest to us share our values [79]. In addition, these distortions intersect with what is often called the “disability paradox.” Non-disabled people, including many clinicians and family members, often systematically underestimate the quality of life that people with significant disabilities report for themselves [80]. Recognizing this paradox is important for UWS decision-making, since it suggests that proxies may interpret DBT-based prognoses that involve survival with serious disability through an unduly pessimistic lens, thereby further complicating, rather than resolving, their moral uncertainty.

## Conclusions

DBTs seem to be a very promising technology for addressing medical uncertainty arising from conditions such as UWS. Owing to the management of large datasets, a DBT can provide informed diagnoses, a clear prognostic path, and serve as a valuable tool for testing drugs or innovative therapies. However, this potentially more precise medical information does not necessarily lead to greater moral certainty. While a DBT can reduce the medical uncertainty surrounding a patient’s condition—by providing a proxy decision-maker with detailed information about diagnosis, prognosis, and possible treatments—it does not resolve the moral uncertainty that arises from value-based judgments. In other words, although DBTs improve the factual basis for decisions, they cannot determine what the proxy ought to do to make an ethically sound choice.

The main reasons are twofold. On the one hand, there is a philosophical and methodological problem: the DBT provides data, but this does not automatically indicate what ought to be done morally. On the other hand, there is a practical decision-making problem: the DBT reduces descriptively-based uncertainty but increases the conflict between moral values and the priorities to be assigned to different factors in order to determine the best interests of the patients. In this context, the surrogate’s choice to withdraw treatment is fundamentally a moral decision, and moral decisions rarely depend solely on empirical and scientific data. Moreover, from a practical perspective, cognitive factors such as biases may influence the proxy’s decision, and the clarity of the information provided may not be sufficient to overcome these cognitive challenges.

We deem that the questions raised herein are not limited to the narrow scope of UWS but may concern other medical contexts in which the patient’s autonomy is questioned, and a surrogate decision-maker is required. Disorders of consciousness, such as coma or minimally conscious state, may pose similar issues as they fall within the same spectrum as UWS; likewise, DBTs could lead to comparable concerns when applied to individuals with intellectual disabilities and psychiatric conditions.

To conclude, while the data provided by a DBT can potentially improve the epistemic position of the proxy decision-maker providing more data about diagnosis, prognosis and treatment recommendations. However, this should be considered a bare minimum, and not the sole justification for our moral choices, since such data do not exempt proxies from navigating complex and difficult ethical considerations.

## Data Availability

No datasets were generated or analysed during the current study.

## Footnotes

1 While the term *vegetative state* is still used in technical contexts such as in the United States [1], it is often considered derogatory and potentially unscientific; we therefore prefer to adopt the term UWS, which has been introduced by scholars in the European Union [2].

## Abbreviations

UWS: Unresponsive wakefulness syndrome
DT: Digital twin
DBT: Digital brain twin
